# BioModels Parameters: a treasure trove of parameter values from published systems biology models

**DOI:** 10.1093/bioinformatics/btaa560

**Published:** 2020-06-23

**Authors:** Mihai Glont, Chinmay Arankalle, Krishna Tiwari, Tung V N Nguyen, Henning Hermjakob, Rahuman S Malik-Sheriff

**Affiliations:** European Molecular Biology Laboratory-European Bioinformatics Institute (EMBL-EBI), Wellcome Trust Genome Campus, Hinxton, Cambridge CB10 1SD, UK; European Molecular Biology Laboratory-European Bioinformatics Institute (EMBL-EBI), Wellcome Trust Genome Campus, Hinxton, Cambridge CB10 1SD, UK; European Molecular Biology Laboratory-European Bioinformatics Institute (EMBL-EBI), Wellcome Trust Genome Campus, Hinxton, Cambridge CB10 1SD, UK; Signalling Department, Babraham Institute, Babraham Research Campus, Cambridge CB22 3AT, UK; European Molecular Biology Laboratory-European Bioinformatics Institute (EMBL-EBI), Wellcome Trust Genome Campus, Hinxton, Cambridge CB10 1SD, UK; European Molecular Biology Laboratory-European Bioinformatics Institute (EMBL-EBI), Wellcome Trust Genome Campus, Hinxton, Cambridge CB10 1SD, UK; European Molecular Biology Laboratory-European Bioinformatics Institute (EMBL-EBI), Wellcome Trust Genome Campus, Hinxton, Cambridge CB10 1SD, UK

## Abstract

**Motivation:**

One of the major bottlenecks in building systems biology models is identification and estimation of model parameters for model calibration. Searching for model parameters from published literature and models is an essential, yet laborious task.

**Results:**

We have developed a new service, BioModels Parameters, to facilitate search and retrieval of parameter values from the Systems Biology Markup Language models stored in BioModels. Modellers can now directly search for a model entity (e.g. a protein or drug) to retrieve the rate equations describing it; the associated parameter values (e.g. degradation rate, production rate, Kcat, Michaelis–Menten constant, etc.) and the initial concentrations. Currently, BioModels Parameters contains entries from over 84,000 reactions and 60 different taxa with cross-references. The retrieved rate equations and parameters can be used for scanning parameter ranges, model fitting and model extension. Thus, BioModels Parameters will be a valuable service for systems biology modellers.

**Availability and implementation:**

The data are accessible via web interface and API. BioModels Parameters is free to use and is publicly available at https://www.ebi.ac.uk/biomodels/parameterSearch.

**Supplementary information:**

Supplementary data are available at *Bioinformatics* online.

## 1 Introduction

Systems biology modelling aims to represent the interaction between biological entities using mathematical formalisms and study emergent behaviour (Le Novère, 2015). Kinetic models of cell, molecular and developmental biology which involve representation of biological processes using differential equations have been shown to divulge mechanistic insight into biological regulation. To build a kinetic model, biological processes, such as cell signalling, metabolism or gene regulation, should be abstracted into a list of reactions with reactants and products. The rates of the reactions are represented mathematically using ordinary, partial or stochastic differential equations (Aldridge *et al.*, 2006). An instrumental and challenging part of this model building process is the identification and estimation of appropriate reaction parameters for model calibration. Searching for model parameters from published literature and models is a crucial, yet laborious task.

Modellers often resort to extensive manual literature mining to retrieve experimentally derived parameter values or experimental data that can be fit to the model to estimate parameters. Alternatively, publicly available curated databases, such as SABIO-RK (Wittig *et al.*, 2018), which provide information specifically on biochemical reactions, their kinetic rate equations with parameter values, are used by the modellers.

To provide an additional avenue to obtain model parameters beyond biochemical reactions, we have developed a new service, the BioModels Parameter search, that can facilitate the search and retrieval of modelled reactions, rate equations and parameter values from published models in BioModels (Malik-Sheriff *et al.*, 2020). BioModels is the world’s largest repository of curated models and is the third most used data resource after PubMed and Google Scholar among the scientists who use modelling in their research (Stanford *et al.*, 2015; Szigeti *et al.*, 2018).

Systems Biology Markup Language (SBML) (Hucka *et al.*, 2019, 2003) is a widely used community format for encoding mathematical models. Having a publicly available specification and open-source software libraries for working with SBML content from many operating systems and programming languages has been instrumental in ensuring that over 250 tools are compatible with the format today. Biological processes can be represented in SBML by means of reactions or equations which describe quantitatively the interplay between the model components. To make this representation less verbose, modellers may define custom functions which can then be referenced in the context of multiple reactions within the model. SBML can also record the biological meaning of the entities in a model in a machine-readable and unambiguous manner, using external cross-references.

Currently, a large proportion of models in BioModels, including all curated ones, are represented in SBML. During the curation process, such models are verified to reproduce the results presented in the corresponding manuscript. This is performed using a different software rather than the one used by the authors. Following curation, models are semantically enriched by cross-linking model entities to external biomedical resources and ontologies.

Leveraging the SBML representation and semantic annotation, we have extracted model entities, reactions, rate equations and associated parameter values from curated and non-curated models and made them searchable via BioModels Parameters. Using the BioModels Parameter search, modellers can now directly look up a model entity (e.g. a protein or drug) to retrieve the associated reactions, along with the rate equations, associated parameter values (e.g. degradation rate, production rate, Kcat, Michaelis–Menten constant in biochemical reactions; rate of cell growth, viral infection or cytotoxic killing for macromolecular processes) and the initial concentrations, which are crucial for building models. Modellers can benefit by retrieving the range of values for a parameter of interest, in order to perform parameter scanning and set initial parameter values for model fitting. The rate equations can also be used to extend an existing model.

This easy-to-use search facility shows for each entity in a model, the reactions in which it is involved, and the corresponding parameter values used by the model authors in a tabular view. Each entry is cross-referenced to the original model and publication, which can be consulted to understand the complete context of the parameter’s usage. Furthermore, model entities are accompanied by links to external resources such as UniProt, ChEBI, GeneOntology, Reactome, or SABIO-RK if they contain additional information. Thus, BioModels Parameters will be a valuable resource for systems biology modellers.

## 2 Method and implementation

**Fig. 1.**
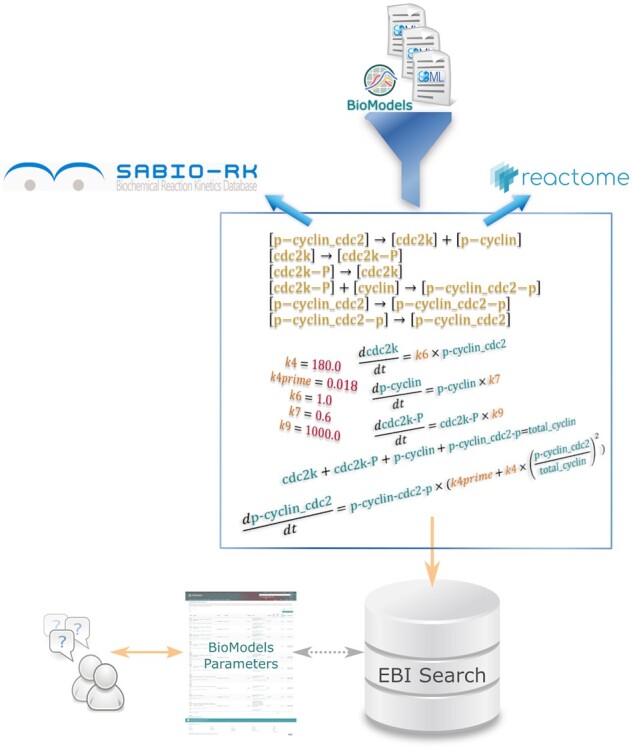
Graphical representation of the methodology employed in this work

We extracted reactions from about 920 curated and 450 non-curated kinetic SBML models described in the literature and publicly available in BioModels. A Java-based tool was created for this purpose, which made use of the JSBML (http://sbml.org/Software/JSBML) (Rodriguez *et al.*, 2015) software library to process the models. Models making use of custom function definitions for reaction rates were converted to semantically equivalent versions with the function definitions inline into the kinetic laws, thus ensuring that the model reactions are only dependent on the biological entities involved in the model and their corresponding parameters. The conversion was performed using libSBML (http://sbml.org/Software/libSBML) (Bornstein *et al.*, 2008). We use the application programming interfaces (APIs) exposed by Reactome (Fabregat *et al.*, 2018) and SABIO-RK to check if the resources contain information about the model entities (aka biochemical species) in the dataset. The BioModels Parameters search results contain links to the appropriate external resources where additional information are available. This workflow, depicted in Figure 1, runs on a regular basis, with the resulting dataset being indexed by the European Bioinformatics Institute (EMBL-EBI) search (Madeira *et al.*, 2019; Park *et al.*, 2017) so that it can be browsed and downloaded either through the BioModels web interface or programmatically.

The source code for the extraction workflow (https://bitbucket.org/biomodels/biomodels-parameters/), as well as the API and the user interface (https://bitbucket.org/biomodels/jummp-biomodels/) are freely available under the terms of the Affero General Public Licence version 3. Documentation about programmatic access to the BioModels Parameter search can be found at https://www.ebi.ac.uk/biomodels/docs/. The BioModels Parameters dataset, just likethe models they originated from, is in the public domain, covered by the Creative Commons CC0 licence.

## 3 Results

### 3.1 BioModels Parameters content

In this work, we extracted parameters from both curated and non-curated kinetic BioModels submissions encoded in SBML and described in the literature. In total about 1,370 kinetic models comprised over 84,000 biochemical reactions involving more than 56,000 entities and 95,000 parameters (Table 1).

**Table 1.** Overview of the number of models and corresponding reactions, entities and parameters available in the BioModels Parameter search

	Curated models	Non-curated models
No. of models	921	454
No. of entities	15,770	40,352
No. of reactions	25,435	58,716
No. of parameters	40,041	55,822

*Note*: Only data from published kinetic models were extracted.

**Fig. 2.**
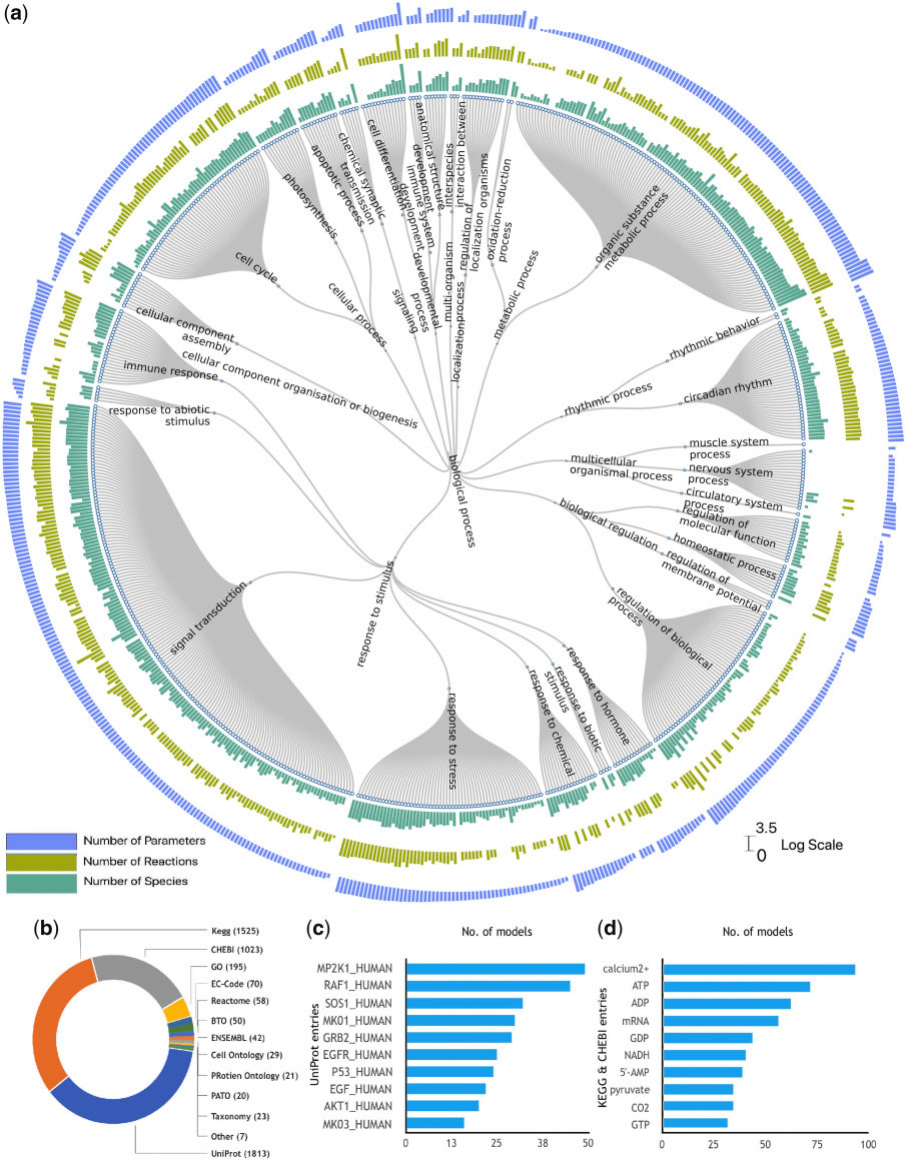
Content statistics for curated models. (**a**) Distribution of biological entities, reactions and parameters available in the BioModels Parameter search grouped by the biological process of the curated model they are defined in. The classification has been created using GO terms from the model level annotation. The end nodes of the dendrogram are individual models. The *Y* axis of the bar plots is represented in logarithmic scale. (**b**) Doughnut-chart illustrating the biological entity cross-references in the BioModels Parameter search grouped by the originating biomedical resource. (**c**) Top 10 UniProt entries referenced by the model entities in the BioModels Parameter search. (**d**) The 10 most frequent metabolites (from KEGG and ChEBI) cross-referenced in the BioModels Parameter search

Reflecting the broad spectrum covered by BioModels submissions, BioModels Parameters provides quantitative information about biological processes including biological regulation, response to stimulus, as well as cellular and developmental processes (Fig. 2a). Thus, BioModels Parameters includes parameters at various scales of space and time, emanating from models of protein conformational change, cell signalling cascades, metabolic pathways, pathogenic infection, cell to cell interaction, tissue level pharmacokinetics and population dynamics. Our resource contains parameters from over 60 different taxa which can be easily filtered in the search and accessed.

The curated models are also semantically enriched by cross-referencing biological entities to various external resources. As a majority of the kinetic models in BioModels represent biochemical reactions, UniProt is the most used resource to annotate model entities, followed by KEGG and ChEBI (Fig. 2b). MAP Kinase (MAPK) cascade is among the most modelled signalling pathways and hence, MAPK is the most frequent protein entity with entries from about 50 models (Fig. 2c). Similarly, calcium signalling and calcium ion are the most modelled system and metabolite, respectively (Fig. 2d).

### 3.2 Accessing data

**Fig. 3.**
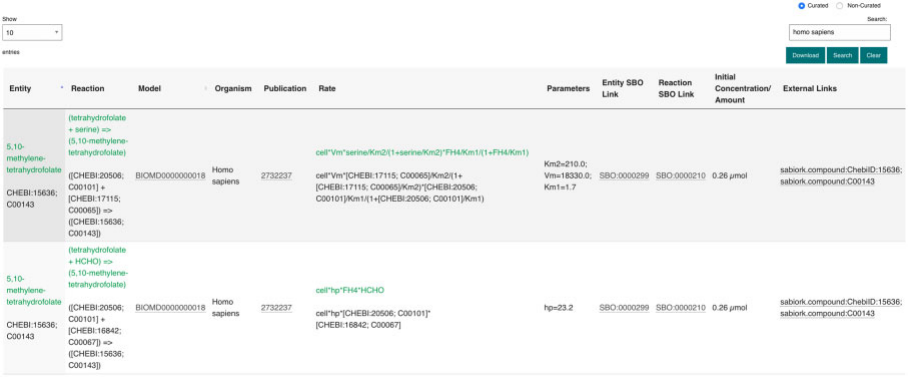
Screenshot of the BioModels Parameter search landing page. Users can view the parameters for every biological entity participating in a reaction defined by a kinetic model hosted in BioModels

BioModels parameter data can be searched and retrieved either through a web browser or programmatically from https://www.ebi.ac.uk/biomodels/parameterSearch/. As shown in Figure 3, available parameters are displayed in tabular format, grouped by the biological entity and reaction they are associated with. For each record, we also display a number of related pieces of information extracted from the originating submission, including the initial amount or concentration of entity, the rate of the reaction, the associated parameter values, as well as the organism and the publication where the model is described.

Moreover, we link to well-established resources such as Reactome (Fabregat *et al.*, 2018) or SABIO-RK when they contain Supplementary Information about a particular biological entity. The model entities are also cross-referenced to the annotated data resource and ontology (Fig. 2b). This allows modellers to ascertain at a glance the parameter value ranges for the biological entity of interest and obtain additional background information about it with a single click. The data can be searched with specific values by using the search field on the right-hand side (Fig. 3). The search results by default include data from manually curated models described in the scientific literature, with an option to include the non-curated ones; this search space can be easily restricted by using the dedicated buttons atop the search box. The advanced search feature allows users to search a keyword specifically within each field such as reactants, parameters, organism and combine them. Users can customise the number of records displayed at any one time, and the result set can be downloaded as a CSV file. Search results can also be bookmarked or shared with collaborators.

Third-party tools can obtain parameter information through a dedicated endpoint in the BioModels REST application programming interface (https://www.ebi.ac.uk/biomodels/docs). The documentation, generated using Swagger, offers an interactive web page that allows to perform sample requests and inspects their corresponding response, thus streamlining the integration of parameter information with third-party software. To maximize interoperability, we support widely used response formats in XML, JSON or CSV, for which dedicated support exists in virtually all mainstream programming languages.

## 4 Use cases

The BioModels Parameter search provides details of all the entities, reactions, rate laws and associated parameters, relevant in creating new models, modifying existing models with new entries and, scanning concentration or parameter ranges to obtain the best fit for the data at hand. Here, we present a few such scenarios where the information extracted from BioModels Parameters supports model development.

### 4.1 Entities concentrations range

**Fig. 4.**
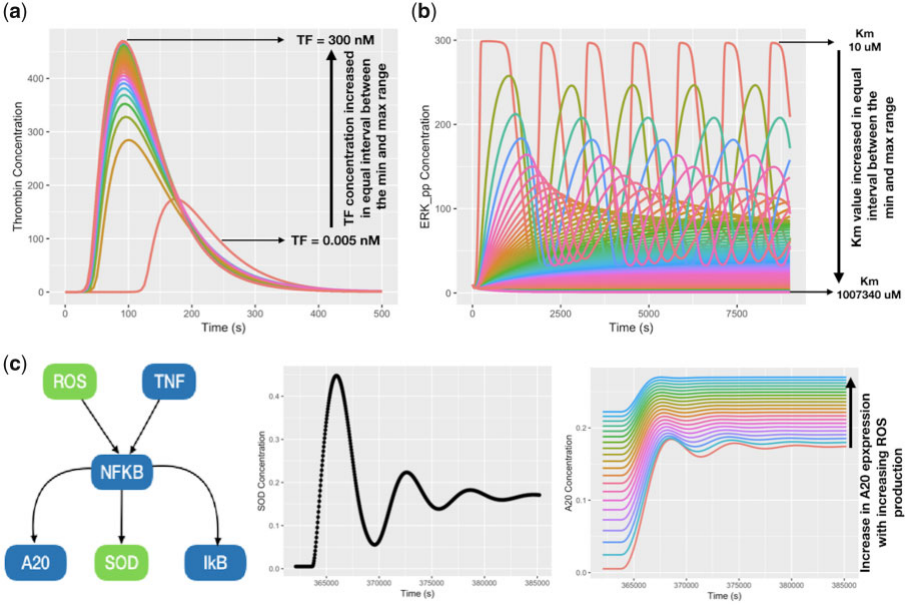
Use cases to demonstrate applications of BioModels Parameters (**a**) Entities concentration range: assessing the effect of various concentrations of TF (0.005–300 nM) on thrombin activation studied in BIOMD0000000332. (**b**) Parameter value range: assessing the effect of various Km (10–1 007 340 µM) on double phosphorylation of ERK by MEK, studied in BIOMD0000000010. (**c**) Model extension: a new model MODEL1911140002 was constructed by extending the TNF-NFkB model BIOMD0000000786 (Lipniacki *et al.*, 2004, blue) from BioModels to study the cross-talk between ROS and NFkB signalling and incorporating new components from BioModels Parameters [extracted from BIOMD0000000560 (Hui *et al.*, 2016, green)]. A subset of model pathway (left), simulation of TNF-induced SOD production (middle) and ROS-induced A20 production (right). Simulation conditions are same as the parent models and only indicated changes in entity concentration and model parameter are scanned. The COPASI representation of the models and their simulation experiment description (SED-ML) files for (a) and (b) that can be used to reproduce the figures are attached as Supplementary Information

Initial levels of the biological entities are needed to run a model simulation. BioModels Parameters allows users to extract the initial concentration values of biological entities from pre-existing models. A modeller can extract and scan a range of values previously used in the literature in order to assess the concentration’s impact on the newly built model. To demonstrate this, we examined the concentration range of the Tissue Factor (TF) protein, a known trigger for extrinsic blood coagulation pathway, and tested its impact on thrombin activation in an existing model [BIOMD0000000332 (Bungay *et al.*, 2006)]. We searched ‘TF’ as keyword in BioModels Parameters. The search results were downloaded and further manually filtered to extract different concentration ranges. We found a TF concentration range from 0.005 to 300 nM used across various models of relevance. We loaded the model BIOMD0000000332 in COPASI (Hoops *et al.*, 2006) and used its ‘Parameter Scan’ feature to run the simulation for the range of TF concentration we had extracted. The output of the scan (Fig. 4a) revealed that increase in TF concentration accentuated the amplitude of thrombin activation as well as decreased the thrombin activation time until respective points of saturation.

### 4.2 Parameter scanning

Identification of kinetic parameter values is one of the most challenging steps in model construction. Although experimentally derived parameters are available in the literature and curated databases such as SABIO-RK, they are scarce. Therefore, kinetic parameters are often estimated fitting the model to relevant data. Any previously estimated parameter value would also be useful for building new models.

Akin to the previous scenario, we used various kinetic parameter values extracted from the parameter search to simulate the model behaviour. We have searched for ‘ERK’ and extracted the values of Michaelis-Menten constant, Km for the extracellular signal-regulated kinase (ERK) phosphorylation reactions across different models, obtaining a range of 10–1,007,340 µM. Applying it to the ERK model BIOMD0000000010 (Kholodenko, 2000), we observed (Fig. 4b) increase in km reduced ERK activation levels as well as changed the ERK dynamics from oscillatory to overdamped regime.

In addition to the above example, parameters can be used as a source of initial values when fitting a model to the experimental data, as seen in BIOMD0000000734 (Parmar *et al.*, 2017).

### 4.3 Adding new components to extend an existing model

In this case, we extended a base model by adding new reactions and parameters from another model so as to study some new biological observations (Fig. 4c). In the base TNF-NFkB signalling model [BIOMD0000000786 (Lipniacki *et al.*, 2004)], Tumar Necrosis Factor (TNF) triggers activation of IκB Kinase (IKK), which degrades Inhibitor of nuclear factor kappa B (IkB) to induce nuclear translocation of NFkB. This, in turn, induces A20 and IkB to control its own activation in a negative feedback manner. To investigate the effect of reactive oxygen species (ROS) on NFkB mediated A20 induction and the role of TNF in production of the detoxifying enzyme, Superoxide Dismutase (SOD), we constructed a new model by adding two components ROS and SOD from another model [BIOMD0000000560 (Hui *et al.*, 2016)] extracted via the BioModels Parameters search. The new model (in SBML format) along with additional files (COPASI and SED-ML files for simulation) was deposited in BioModels (Malik-Sheriff *et al.*, 2020) and assigned the identifier MODEL1911140002. In our model, we added a new trigger in the system, ROS, which is known to activate NFkB by inducing IkB degradation and subsequent SOD production via NFkB. Our model simulation revealed that NFkB activation increased with the ROS level, which consequently increased the production of A20. Similarly, TNF also induced SOD production by inducing NFkB activation. Thus, equations and parameters from two different models can be combined to study the response to new triggers in the system.

## 5 Discussion

We here present BioModels Parameters, a service which streamlines the search and retrieval of parameter information from kinetic models. Identifying the appropriate rate equations, parameter values and initial entities concentration are the key parts of the model development process, and these tasks often require significant effort searching the literature. Modellers can significantly reduce this overhead by searching the BioModels Parameters dataset instead. An inevitable limitation is that the parameter search is contingent on depositions available in the BioModels repository.

The components emerging from curated models are more reliable than those from the non-curated ones, as the former are independently verified to be reproducible. Nevertheless, the model parameters are not individually curated to ascertain whether they are derived from experiments or estimated. The users should refer to the corresponding publications to understand the complete context of parameter usage and model assumptions. However, the parameter search provides a quick overview of available data and possible range. The cross-linking to resources such as UniProt, Gene Ontology (GO), ChEBI, Reactome or Sabio-RK enables users to quickly access more relevant information and establish the relevance of the available data to their modelling building exercise.

In summary, we retrieved published models from BioModels and broke them down into reusable fundamental blocks including the reactions, rate equations and the parameters; made them easily searchable and provided cross-references so that they can be used to build new models or extend existing ones. Thus, BioModels Parameters demonstrates the value of standards in model representation and curation. We can only extract parameters from about 1,370 models and make them accessible for systematic re-use because they are represented and curated according to common community guidelines. BioModels Parameters, as a service to search and access reusable model components, can greatly benefit scientists who build and use models in their research.

## Acknowledgements

The authors would like to acknowledge the support of SABIO-RK, Reactome and EMBL-EBI Search Teams.

## Funding

This work has received funding from the Innovative Medicines Initiative 2 Joint Undertaking under grant agreement number 116030. This Joint Undertaking receives support from the European Union’s Horizon 2020 Research and Innovation Programme and European Federation of Pharmaceutical Industries and Associations (EFPIA). This work has also been supported by the Biotechnology and Biological Sciences Research Council (BBSRC) [BB/N019482/1, BB/N019474/1 (MultiMod)] and European Molecular Biology Laboratory (EMBL).

Funding for open access charge: Research Councils UK Open Access.

*Conflict of Interest*: none declared.

## Supplementary Material

btaa560_Supplementary_Files
